# Whole genome DNA methylation profiles define Meniere’s disease subclusters

**DOI:** 10.1007/s00109-025-02581-6

**Published:** 2025-08-06

**Authors:** Vibha Patil, Pablo Cruz-Granados, Francisca E. Cara, Juan Carlos Amor-Dorado, Ismael Aran, Andres Soto-Varela, Patricia Perez-Carpena, Jose Antonio Lopez-Escamez

**Affiliations:** 1 Meniere’s Disease Neuroscience Research Program, Faculty of Medicine & Health, School of Medical Sciences, The Kolling Institute, The University of Sydney, Sydney, NSW Australia; 2 Otology & Neurotology Group CTS495, Division of Otolaryngology, Department of Surgery, Instituto de Investigación Biosanitaria, ibs.GRANADA, Granada, Universidad de Granada, Granada, Spain; 3 Sensorineural Pathology Programme, Centro de Investigación Biomédica en Red en Enfermedades Raras, CIBERER, Madrid, Spain; 4 Department of Otolaryngology, Hospital Can Misses, Ibiza, Spain; 5 Department of Otorhinolaryngology, Complexo Hospitalario Universitario de Pontevedra, Pontevedra, Spain; 6 Division of Neurotology, Department of Otorhinolaryngology, Complexo Hospitalario Universitario, Santiago de Compostela, Spain; 7 Department of Surgery and Medical-Surgical Specialties, Universidade de Santiago de Compostela, Santiago de Compostela, Spain; 8 Health Research Institute of Santiago, Santiago de Compostela, Spain; 9 Department of Surgery and Medical-Surgical Specialties, University of Granada, Granada, Spain; 10 Department of Otorhinolaryngology, Hospital Universitario de Granada, Granada, Spain

**Keywords:** WGBS, Meniere’s disease, T-cells, Immune profile, DNA methylation, Hearing loss

## Abstract

**Abstract:**

Meniere disease (MD) is a cochleo-vestibular syndrome defined by episodes of vertigo associated with tinnitus and sensorineural hearing loss. While MD immune response has been linked to autoinflammation and type 2 cytokines, other molecular mechanisms such as DNA methylation have an emerging yet underexplored role in MD pathophysiology.To understand the role of DNA methylation in MD, we performed whole-genome bisulphite sequencing in MD patients (*n* = 40) and controls (*n* = 13) and used differentially methylated cytosines (DMCs) to define clusters, cell types, and biochemical pathways in MD. We found three MD subclusters: Cluster 1 (40% of patients) and Cluster 3 (25%) showed DMC profiles against controls, while Cluster 2 (35%) did not. Significant DMCs from Cluster 1 and Cluster 3 versus Control analysis were annotated to 3033 and 59 unique genes, respectively. Each cluster showed a different gene enrichment; however, the *KDMB4* gene had significant upregulated DNA accessibility in a complementary ATAC-seq dataset and showed significant DMCs in both Cluster 1 and Cluster 3. DNA methylation patterns in MD reveal three clusters which are reflective of an underlying difference in pathways related to cytokine stimulus, immunity T-cell, and NK-cell pathways. *KDMB4* emerges as a critical MD gene which deserves further research.

**Key messages:**

- We asked if DNA methylation can help understand Meniere’s Disease (MD) pathophysiology.
- DNA methylomes group MD patients into three distinct sub-clusters.
- DNA methylation in MD reflect difference in pathways related to neurons and cytokine stimulus.
- The data shows *KDMB4* emerging as a key gene that requires further multi-modal investigation.

**Supplementary Information:**

The online version contains supplementary material available at 10.1007/s00109-025-02581-6.

## Introduction

Meniere disease (MD) is a debilitating syndrome defined by episodes of vertigo associated with tinnitus and sensorineural hearing loss (SNHL) with a significant impact on patients’ health-related quality of life [1]. It has a significant genetic contribution with several genes in the tectorial membrane and hair cell stereocilia links segregating in multiple families with autosomal dominant and recessive inheritance [2]. Most MD cases, however, occur sporadically with growing evidence supporting a central role of patient immune response and MD subtype. For example, MD patients exhibited a persistent systemic inflammation associated with alterations in immune response, including high levels of proinflammatory cytokines [3, 4], comorbid allergy [5, 6], autoimmunity [7], and autoinflammation [8] according to collective evidence from epigenomic [9], transcriptomic [10], and single-cell proteomic studies [11].

Methylation of cytosines in DNA is an epigenetic mechanism known to have significant impacts on transcriptional regulation of the genome in a variety of disease processes. The frequency and genomic distribution of DNA methylation aid in coordinating expression of gene pathways in concert with other layers of epigenetic regulators such as histone modifications [12]. Hence, both direct and indirect transcriptional consequences may occur based on the density and genomic distribution of DNA methylation occurrence. Emerging evidence suggests that DNA methylation may be implicated in MD pathophysiology. For example, a WGBS study in human blood samples taken from sporadic cases of MD patients and healthy controls (case = 14, control = 6) revealed patients with varying levels of IL-1β also presented with distinct, global DNA methylation patterns [9]. However, the sample size was highlighted as a limitation of this study, and further validation with larger cohorts was required.

The goal of this study was to assess global DNA methylation patterns using WGBS datasets from a larger PBMC sample set of MD patients and healthy controls compared to previous studies. We also aimed to elucidate potential gene pathways relevant to MD pathophysiology that were likely to be affected by aberrant DNA methylation. We obtained WGBS data of blood samples of a total of 53 individuals (40 cases and 13 controls) from various regions of Spain. Our analysis revealed that global DNA methylation patterns categorize MD patients into three distinct clusters. The finding suggests that these clusters may be defined predominantly by an underlying difference in inflammatory profiles and T-cell functions.

## Results

### DNA methylation defines subclusters in Meniere’s disease

**Fig. 1:**
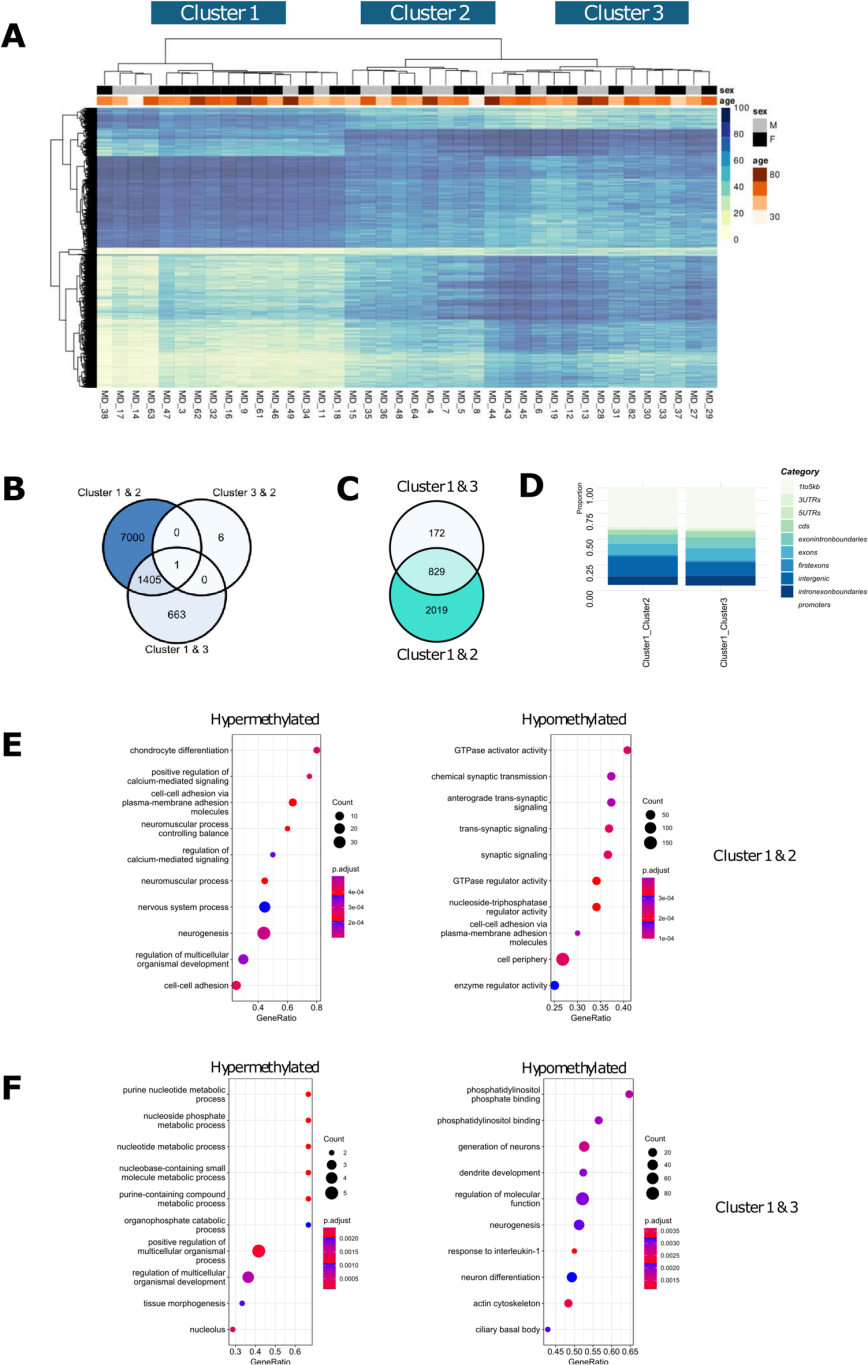
Meniere’s disease subjects cluster into three distinct groups. **A** Heatmap showing unsupervised clustering of significant DMCs (%) showing three distinct clusters. Age and gender distribution across clusters is indicated by the horizontal bars above the heatmap. **B** A Venn diagram showing the total, overlapping, and unique DMCs that were detected in each differential methylation analysis conducted between the three MD clusters. **C** A Venn diagram showing the total, overlapping, and unique DMCs which were annotated to genes. **D** A comparison of total counts of significant DMCs detected across annotated gene regions between each grouped analysis. Gene Ontology enrichment analysis of a weighted gene list based on annotated DMCs detected in the comparison **E** between Cluster 1 and Cluster 2 and **F** between Cluster 1 and Cluster 3. Gene pathways associated with hyper- and hypo-methylated DMCs are present on the left and right, respectively. The top ten most significant pathways are shown

The methylation profiles of the 40 MD case–control cohort (*n* = 40) were subjected to unsupervised hierarchical clustering using the Ward.D method, which identified three distinct clusters (Fig. 1A). Cluster 1 was the largest group (*n* = 16) encompassing 40% of the total number of samples. Details regarding familial history, candidate mutations, and age range of each cluster are summarized in Table 1. Both age and gender distribution of samples could not explain the clustering as indicated by the heatmap (Fig. 1A). To understand the differences between clusters further, differential methylation was assessed between each cluster, by comparing the number of overlapping differentially methylated cytosines (DMCs). The largest difference was observed between Cluster 1 and Cluster 2 with 8405 DMCs identified (Fig. 1B) which were annotated (± 100 bp of gene region) to 2019 unique genes (Fig. 1C). A small number of DMCs (*n* = 7) between Cluster 2 and Cluster 3 suggest there is very little difference in methylation patterns between these clusters. However, since a large number of DMCs (*n* = 633) were observed between Cluster 1 and Cluster 3, this suggests that Cluster 3 has a unique methylation pattern from other clusters (Fig. 1B). The 663 DMCs were annotated to 172 unique genes (Fig. 1C). We then looked at whether there were any differences regarding the genomic distribution of these DMCs between clusters since the genomic location of differential methylation has various implications in genomic regulation. No significant difference was observed between the proportional distribution of DMCs from each analysis across various gene regions. However, the largest proportion of DMCs for both clusters was within gene regions defined as “intergenic regions” and “1 to 5 kb from transcription start sites (TSS)” (Fig. 1D). Genes with significant DMCs were then ranked based on the degree and frequency of methylation per gene (according to the equation defined in the Methods) and subsequently assessed for GO enrichment analysis (GSEA). Genes annotated to hypermethylated and hypomethylated DMCs, which defined the differences between Cluster 1 and Cluster 2, were enriched for pathways primarily related to cell adhesion, cell signaling, and calcium signaling (Fig. 1E). Genes annotated to hypermethylated DMCs, which defined the difference between Cluster 1 and Cluster 3, were enriched for pathways primarily related to metabolic processes. Genes annotated to hypomethylated DMCs, which defined the difference between Cluster 1 and Cluster 3, were enriched for pathways primarily related to neuronal development and interleukin 1 signaling (Fig. 1F). Together, these results show that the three MD clusters are distinct with unique DMCs and molecular features.

**Table 1:** Summary statistics of age, % familial MD cases, gender distribution, and previous existing mutations for each cluster resulting from the initial unsupervised clustering of all samples

	Cluster 1	Cluster 2	Cluster 3	Control
Sex (% female)	66.7	45.5	57.1	61.5
Age Min	26	29	42	25
Age (mean ± SD)	57.9 ± 14.2	51 ± 13.5	58.4 ± 12.4	43.2 ± 13.2
Age Max	79	76	81	61
FMD (*n*)	0	1	1	NA
Mutations detected	None	Not Found	1 in *TECTA* (chr11:121036077C > T)	NA

### Observed DNA methylation profiles are predominantly related to T-cells and NK-cells

Given that PBMC isolation can yield a mixed population of cells, we used a deconvolution method as described previously [13], to identify the cell-type composition of each sample, based on a library of reference methylation profiles for individual cell types. Deconvolution analysis confirmed the presence of heterogeneous blood cell types across samples; however, this variability did not correspond with or explain the clustering structure, indicating that cell-type heterogeneity is unlikely to be the primary driver of the observed methylation-based clustering. All samples had a heterogeneous cell-type composition with gastric and ovarian epithelium cells contributing large portions in some samples (Supplementary Fig.  1 A). The relative proportion of blood cells represented within each MD cluster and the Control group was also assessed (Supplementary Fig. 1B). All samples showed some representation of T-cells, NK-cells, and megakaryocytes; however, a high degree of variability was observed in the presentation of other blood cell types, especially monocytes and B-cells (Supplementary Fig. 1B). However, unsupervised clustering showed that the three distinct MD sample clusters identified previously were not attributable to variability in cell-type composition (Supplementary Fig.  1 A). These findings suggest that the patient clusters identified were not likely to be driven by heterogeneity in cell types between samples.

### MD subclusters show distinct differences to healthy controls

**Fig. 2:**
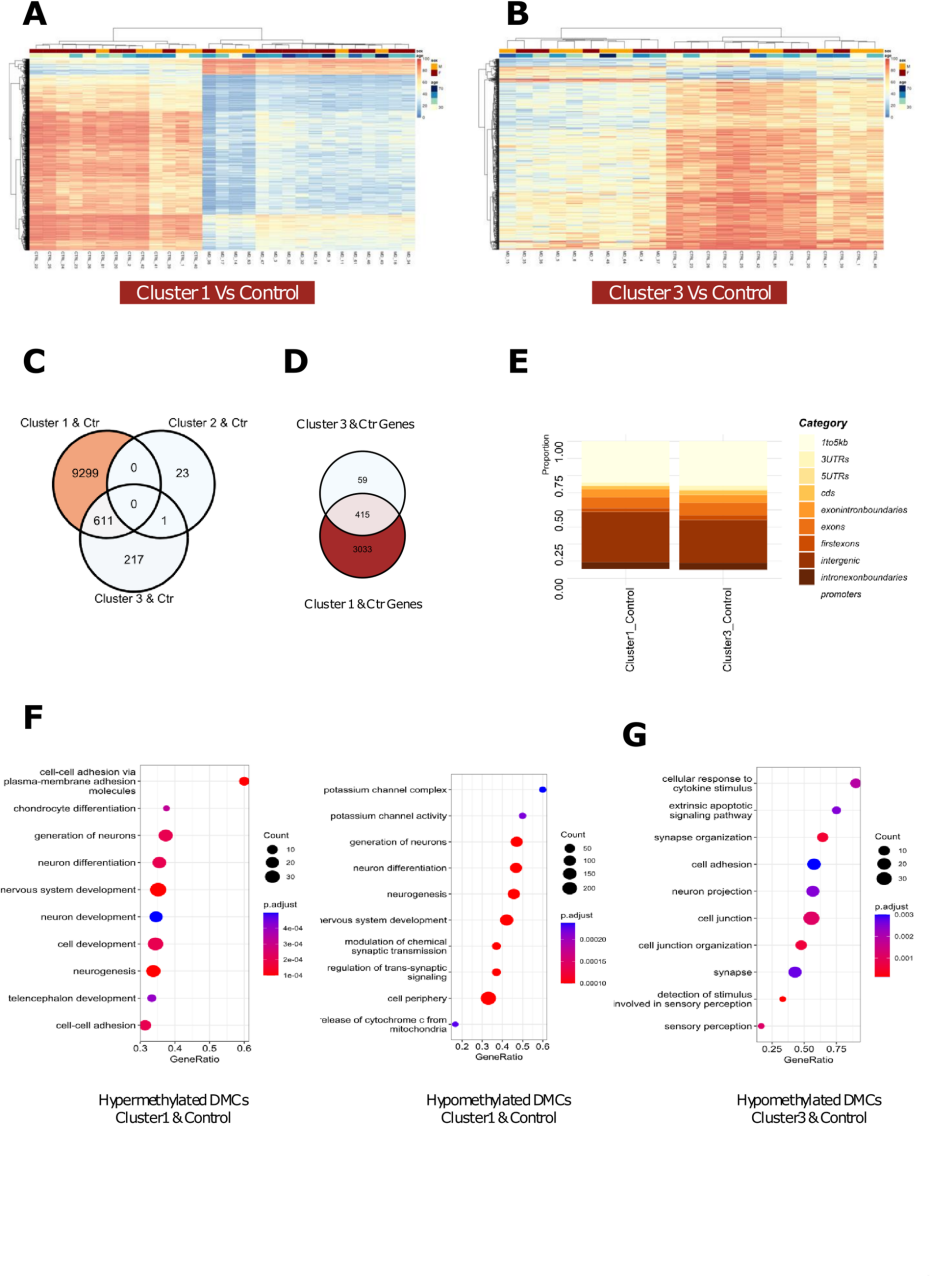
Meniere’s disease methylomes are distinct from healthy controls. **A** Heatmap showing unsupervised clustering of methylation values of the top 2000/9911 significant DMCs identified between MD Cluster 1 and Control samples. Age and gender distributions across samples are indicated by the horizontal bars above the heatmap. **B** Heatmap showing unsupervised clustering of methylation values of all 829 significant DMCs identified between MD Cluster 3 and Control samples. Age and gender distributions across samples are indicated by the horizontal bars above the heatmap. **C** A Venn diagram showing the total, overlapping, and unique DMCs that were detected in each differential methylation analysis conducted between the three MD clusters and the control group. **D** A Venn diagram showing the total, overlapping, and unique DMCs which were annotated to genes detected in each differential methylation analysis conducted between the three MD clusters and the control group. **E** A comparison of total counts of significant DMCs detected across annotated gene regions between each grouped analysis. **F** Gene ontology enrichment analysis of a weighted gene list obtained from DMCs annotated from the analysis of Cluster 1 versus Control. Gene pathways associated with hypermethylated DMCs are present on the left, while gene pathways associated with hypomethylated DMCs are present on the right. The top ten most significant pathways are shown. **G** Gene ontology enrichment analysis of a weighted gene list obtained from DMCs annotated from the analysis of Cluster 3 versus Control. Gene pathways associated with hypomethylated DMCs are present on the right. The top ten most significant pathways are shown

To determine whether DNA methylation patterns differentiate MD patients from healthy controls, differential methylation was conducted between each MD cluster and the Control group. Unsupervised clustering of the top 2000 significant DMCs between Cluster 1 and Control (Fig. 2A), as well as between Cluster 3 and Control (Fig. 2B), revealed that the observed differential methylation separating these two groups was not likely to be driven by age or gender.

A total of 9910 DMCs were identified between Cluster 1 and Control groups, with 67.6% (*n* = 6702) of these DMCs annotated to be within ± 100 bp of a gene. In addition, 6.7% (*n* = 665) of these genes were microRNAs or long non-coding RNAs. Similarly, 829 DMCs were identified between Cluster 3 and Control groups, with 71.1% (*n* = 590) of these DMCs annotated to be within ± 100 bp of a gene. In addition, 9.1% (*n* = 55) of these genes were microRNAs or long non-coding RNAs. Of the 24 DMCs shared between Cluster 2 and Control, 23 were unique to this pairing, while one DMC was also shared with Cluster 3 (Fig. 2C). Significant DMCs from Cluster 1 versus Control and Cluster 3 versus Control analysis were annotated to 3033 and 59 unique genes, respectively, while 415 genes were common to both groups (Fig. 2D). The largest proportion of DMCs for both Cluster 1 versus Control and Cluster 3 versus Control comparisons were annotated to “intergenic regions” and “1 to 5 kb from TSS” (Fig. 2E). Genes with significant DMCs were then ranked based on the formula described in the Methods section and then assessed for GO enrichment analysis.

Genes annotated to both hypermethylated and hypomethylated DMCs, which define the differences between cluster 1 and controls, were primarily enriched for pathways related to cell–cell adhesion via plasma-membrane adhesion, potassium channels, and mitochondrial function (Fig. 2F). Genes annotated to hypermethylated DMCs, which define the difference between cluster 3 and controls, however, were not associated with any enriched pathways, while genes with hypomethylated DMCs in this comparison were significantly enriched for cytokine stimulation, cell adhesion, and neuron synapse pathways (Fig. 2G). These results indicate that only Clusters 1 and 3 were significantly differentiated from the Control group based on methylation patterns, rather than phenotypic features such as age or gender. The methylation profile of Cluster 2, on the other hand, did not show any distinct differences compared to the Control group, suggesting the need for further investigation into other molecular features.

### Identifying potential molecular differences between Cluster 1 and Cluster 3 MD patients

**Fig. 3:**
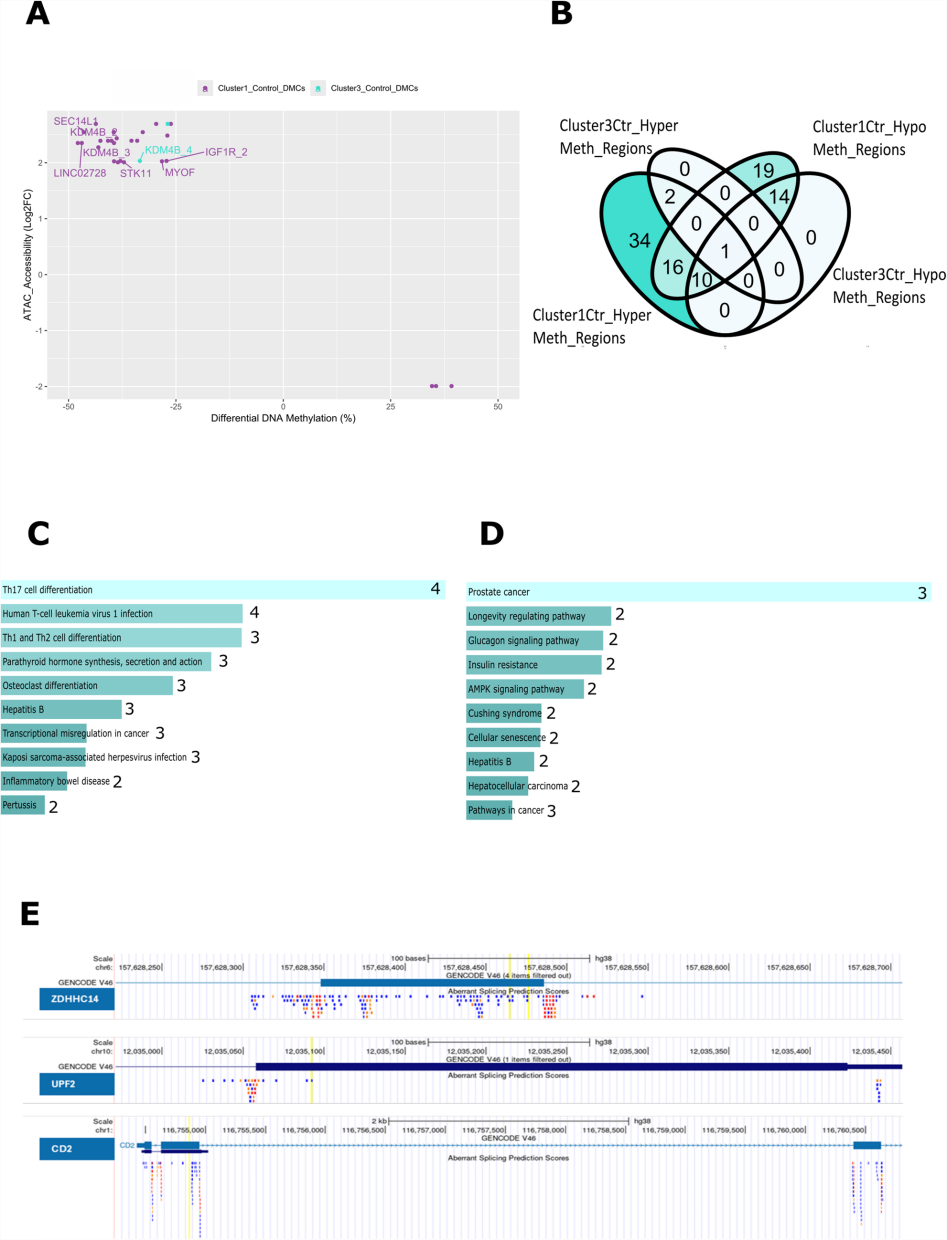
DMC Overlaps with DNA accessibility sites and splice sites. **A** Scatterplot indicating the methylation differences (%) (*x*-axis) of significant DMCs and corresponding log2FC of ATAC-seq accessibility regions (*y*-axis) that were found significant in ATAC-seq analysis [14]. DMCs detected from the Cluster 1 versus Control are indicated in purple, whereas DMCs of the Cluster 3 versus Control are indicated in blue. **B** Venn diagram showing overlapping TFs associated with TF motif enrichment obtained from Homer for all hypermethylated DMC regions from Cluster 1 versus Control, hypermethylated DMC regions from Cluster 3 versus Control, hypomethylated DMC regions from Cluster 1 versus Control as well as hypomethylated DMC regions from Cluster 3 versus Control. Bar plot indicating KEGG enrichment pathways for transcription factors unique to **C** hypermethylated and **D** hypomethylated DMC regions from Cluster 1 versus Control (*n* = 34). **E** UCSC screenshots showing significant DMC locations (highlighted in yellow) which overlapped with known HEX Event splice sites were overlaid on UCSC hg38 database. ZDHC14, UPF2, and CD2 gene regions have aberrant splice site prediction directly overlapping significant DMCs detected in these genes

To evaluate whether Cluster 1 or Cluster 3 MD subtypes could be associated with previously defined molecular phenotypes of MD, we utilized a pseudo-bulk analysis from single-cell ATAC seq datasets from MD patient samples (case = 1634 cells, control = 7557 cells) with a known predisposition for IL-1β overexpression [14]. We first assessed whether any genomic regions of significant differential DNA accessibility identified from this data overlapped with any significant DMCs from both Cluster 1 versus Control and Cluster 3 versus Control as summarized in Table 2 (Fig. 3A). Of the 31 DMC locations which overlapped ATAC-Seq data, only 2/31 were from Cluster 3 versus Control. In addition, 9/31 sites were annotated to protein coding gene regions while the rest were annotated to microRNAs or long non-coding RNAs. Only one genomic region was common to Cluster 1 versus Control and Cluster 3 versus Control. This region, Chr19:4976931, was annotated to the *KDMB4* gene. Increased DNA accessibility compared to control with concurrent hypomethylated CpG sites in the *KDMB4* gene is suggestive of overexpression of this gene in all MD cases versus controls irrespective of MD cluster (Fig. 3A).

**Table 2:** List of DMCs which were found to overlap significantly upregulated genomic regions in complementary ATAC-Seq dataset [1]

Location	*p*-value	*q*-value	Methylation (%)	Status	Gene
Cluster 1 DMCs overlapping ATAC_Seq regions					
chr19.7674882.7674882	1.31*E* − 22	1.65*E* − 19	32.73	hypo	
chr17.77100293.77100293	9.72*E* − 22	1.03*E* − 18	46.46	hypo	*SEC14L1*
chr19.7674709.7674709	7.02*E* − 21	6.27*E* − 18	39.48	hypo	
chr19.7674712.7674712	2.63*E* − 20	2.08*E* − 17	39.47	hypo	
chr2.8598865.8598865	8.39*E* − 19	5.01*E* − 16	38.79	hypo	
chr3.194527150.194527150	1.46*E* − 18	8.32*E* − 16	40.82	hypo	
chr15.98788804.98788804	8.88*E* − 17	3.59*E* − 14	53.00	hypo	*IGF1R*
chr19.4976931.4976931	6.20*E* − 15	1.79*E* − 12	52.25	hypo	*KDM4B*
chr8.115846121.115846121	3.12*E* − 13	6.61*E* − 11	43.07	hypo	
chr3.194527225.194527225	6.03*E* − 13	1.22*E* − 10	29.60	hypo	
chr1.226035089.226035089	4.57*E* − 12	7.84*E* − 10	39.42	hypo	
chr11.78424649.78424649	4.60*E* − 12	7.87*E* − 10	46.92	hypo	*LINC02728*
chr3.194527540.194527540	4.94*E* − 12	8.43*E* − 10	43.65	hypo	
chr16.30551768.30551768	5.35*E* − 11	7.49*E* − 09	35.56	hyper	
chr19.4976895.4976895	9.26*E* − 11	1.23*E* − 08	42.58	hypo	*KDM4B*
chr15.98788891.98788891	1.29*E* − 10	1.67*E* − 08	27.19	hypo	*IGF1R*
chr19.4976921.4976921	1.32*E* − 10	1.71*E* − 08	37.92	hypo	*KDM4B*
chr3.194527532.194527532	2.44*E* − 10	3.00*E* − 08	38.51	hypo	
chr19.1180949.1180949	2.59*E* − 10	3.16*E* − 08	37.10	hypo	*STK11*
chr1.226034950.226034950	2.26*E* − 09	2.26*E* − 07	47.84	hypo	
chr1.226035070.226035070	2.46*E* − 09	2.44*E* − 07	35.36	hypo	
chr16.30551760.30551760	3.87*E* − 09	3.69*E* − 07	39.17	hyper	
chr1.226035143.226035143	8.40*E* − 09	7.42*E* − 07	34.02	hypo	
chr15.90830815.90830815	1.25*E* − 08	1.06*E* − 06	39.99	hypo	
chr3.194527483.194527483	1.88*E* − 07	1.18*E* − 05	26.18	hypo	
chr16.30551721.30551721	2.32*E* − 07	1.42*E* − 05	34.62	hyper	
chr12.131860534.131860534	3.77*E* − 07	2.17*E* − 05	27.00	hypo	
chr20.830253.830253	8.98*E* − 07	4.59*E* − 05	39.41	hypo	
chr10.93427696.93427696	7.28*E* − 05	0.001774	28.26	hypo	*MYOF*
Cluster 3 DMCs overlapping ATAC_Seq regions					
chr3.194527150.194527150	4.06*E* − 07	0.000645	26.92	hypo	
chr19.4976931.4976931	1.16*E* − 05	0.007498	33.41	hypo	*KDM4B*

In addition, to assess potential molecular pathways that may be unique to each MD patient cluster, we assessed whether genomic regions ± 200 bp of significant hyper- and hypomethylated DMCs from both Cluster 1 versus Control and Cluster 3 versus Control were enriched for transcription factor motifs using homer (Fig. 3B).We observed 34 transcription factors associated with enriched motifs that were unique to hypermethylated DMCs from the Cluster 1 versus Control group, and 19 transcription factors associated with enriched motifs that were unique to hypomethylated DMCs from the Cluster 1 versus Control (Supplementary Table 1). For preliminary validation, the top three TFs from this analysis, which contained hypermethylated DMCs in Cluster 1, were then assessed for relative expression. Three arbitrarily selected RNA samples from Cluster 1 and two RNA samples from the Control group were selected (Supplementary Fig.  3 A). As anticipated, these TFs, LEF1, IRF2, and IRF3, were expressed consistently lower in Cluster 1 compared to Control group (Supplementary Fig.  3 A). No unique TFs were identified for Cluster 3 versus Control (Fig. 3B). TFs associated with hypermethylated DMC regions from Cluster 1 versus Control and Cluster 3 versus Control were further analyzed for KEGG pathway enrichment using the EnrichR database. Notably, T-cell pathways related to Th1, Th2, and Th17 responses were highlighted as the most significant in the TF gene set from Cluster 1 versus Control group (Fig. 3C). In contrast, non-specific pathways were highlighted from the TF gene set associated with Cluster 3 versus Control group (Fig. 3D).

### Several DMCs overlap splice sites in Cluster 1 and 3 MD patients

Significant DMCs from both Cluster 1 versus Control as well as Cluster 3 versus Control analyses were then assessed for whether their genomic locations overlapped with known transcriptional splice site regions, as summarized in Table 3. DMCs of the Cluster 1 versus Control were found to overlap with 8 splice sites while only 1 splice site overlapped with DMCs detected from the Cluster 3 versus Control group. Of all hypomethylated DMCs overlapping splice sites, the DMC associated with the splice site of *UPF2* gene was the most significant (*p*-value < 1.08*E* − 37), with the greatest difference in methylation (40.4%) observed from the Cluster 1 versus Control. On the other hand, of all hypermethylated DMCs overlapping splice sites, the DMC associated with the CD2 splice site was the most significant (*p*-value < 4.63*E* − 10) with the greatest difference in methylation (40.6%) observed from the Cluster 1 versus Control. When cross-referenced with the UCSC database, 3/9 of these sites were found to directly overlap with aberrant splice site predictions (Fig. 3E). Since DNA methylation is known to impede TF binding, the presence of these DMCs across the 7 genes identified, especially for *CD2* and *UPF2* from Cluster 1 versus Control, may present an important mechanism of transcriptional regulation that distinguishes MD Cluster 1 from MD Cluster 3 patient subgroups, which warrants further attention.

**Table 3:** List of DMCs which were found to overlap known splice sites

Chr	Splice site	*p*-value	Methylation (%)	Gene	Status	Group
chr1	116,754,866	4.63*E* − 10	40.6091	*CD2*	hyper	Cluster1_Ctr
chr5	88,823,763	4.61*E* − 09	32.0743	*MEF2C*	hypo	Cluster1_Ctr
chr6	157,628,477	8.81*E* − 13	44.0251	*ZDHHC14*	hypo	Cluster1_Ctr
chr6	157,628,465	9.19*E* − 08	26.4138	*ZDHHC14*	hypo	Cluster1_Ctr
chr6	7,579,740	2.91*E* − 07	30.9891	*DSP*	hype	Cluster1_Ctr
chr6	7,579,697	2.7*E* − 06	33.8222	*DSP*	hype	Cluster1_Ctr
chr10	12,035,093	1.08*E* − 37	40.427	*UPF2*	hypo	Cluster1_Ctr
chr10	28,610,784	6.67*E* − 13	36.5859	*WAC*	hypo	Cluster1_Ctr
chr12	92,779,000	1.17*E* − 05	27.97178131	*EEA2*	hypo	Cluster3_Ctr

### DMC overlaps with hearing loss genes, stria vascularis genes, and axonal guidance genes

Next, we compared genes annotated to significant DMCs from both Cluster 1 versus Control and Cluster 3 versus Control groups with known genes from the SNHL database (Supplementary Table 3). While the Cluster 1 versus Control group showed DMCs within 25 hearing loss genes, the Cluster 3 versus Control group showed DMCs within only 1 hearing loss gene (Supplementary Fig.  2 A). Only *DMXL2* was annotated to significant DMCs from both Cluster 1 versus Control (4 sites, avg methylation =  − 34.3% ± 8.8) and Cluster 3 versus Control (2 sites, avg methylation =  − 30.12% ± 0.57) (Supplementary Fig.  2 A). These genes were then assessed against previous studies which have reported significant differential methylation in hearing loss genes of MD patients. Any genes from our study which overlap with previous studies are highlighted in Supplementary Table 2. Similarly, genes from both groups overlapped with a human stria vascularis gene set identified previously from a meta-analysis [15]. Cluster 1 versus Control group showed DMCs uniquely located within 103 SV genes while Cluster 3 versus Control group showed DMCs uniquely located within 4 SV genes (Supplementary Fig. 2B, Supplementary Table 3). Finally, we overlapped genes annotated to significant DMCs from both groups to known genes from a curated list known to affect axonal guidance in MD (Supplementary Table 4). While Cluster 1 versus Control group showed DMCs within 51 genes unique to this group, Cluster 3 versus Control group showed no gene overlaps (Supplementary Fig.  2 C).

## Discussion

Global DNA methylation profiles in MD are distinct from healthy controls and identify potential clusters based on unique molecular features, which warrant further investigation. Differential DNA methylation associated with chromatin accessibility, TF binding motifs, and alternative splicing suggests underlying differences in molecular features related to important MD biological pathways, including neuronal development, neuronal synapses, and immunity-related pathways. We found differentially methylated CpG sites within our patient cohort significantly enriched for unique gene sets. DMCs found in cluster 1, when compared to cluster 3 and control groups, were enriched within genes primarily involved in neuronal development, synaptic signaling, as well as calcium and potassium channel signaling. DMCs found in Cluster 3, when compared to both Cluster 1 and controls, seem to be enriched within genes that are primarily involved in cell adhesion, cell junction, metabolic processes, as well as cytokine-related pathways. Unlike other groups, Cluster 2 DNA methylation patterns did not differ significantly from controls. Overall, DNA methylation profiles provide additional evidence that MD could be categorized into subclusters based on underlying molecular phenotypes.

Previous studies have reported an overload of damaging missense variants in genes related to axonal guidance signaling in MD [15]. Comparing genes annotated to significant DMCs identified from MD clusters versus Control against the reference gene set related to axonal guidance signaling [15], we found 37 of these genes to uniquely overlap with genes from Cluster 1 versus Control (Supplementary Table 4, Supplementary Fig. 2D). Notably, we did not observe any unique overlaps with Cluster 3 versus Control group, suggesting that axonal guidance-related genes are more likely to be affected by differential methylation in Cluster 1. In support of this, among the 103 unique overlaps observed within Cluster 1 versus Control genes with DMCs and SV genes (Supplementary Fig. 2B), axon guidance was the most significantly enriched KEGG pathway identified alongside leukocyte migration and chemokine signaling (Supplementary Fig.  2 C). Interestingly, “response to interleukin 1” pathway was enriched among hypomethylated DMCs between Cluster1 versus Cluster 3. All DMCs within the interleukin 1 gene in Cluster 1 versus Control DMCs were hypomethylated, while no DMCs were detected within Cluster 3 versus Control. Of note, 1 out of 4 of the hypomethylated DMCs detected in Cluster 1 versus Control group is localized to the TSS of a specific interleukin 1 transcript. Hence together, this suggests Cluster 1 subjects may display a molecular profile that is more pronounced in variability in synaptic signaling, axonal guidance, calcium signaling, as well as potential alternative transcription of interleukin 1 variants compared to Cluster 3 subjects.

In order to further identify potential molecular differences between Cluster 1 and Cluster 3 MD subgroups, TF enrichment was assessed for all regions within ± 200 bp of significant DMCs from both groups. Only Cluster 1 showed unique TFs that were associated with both hyper- and hypomethylated DMC regions. Furthermore, qRT-PCR of TFs containing hypermethylated DMCs unique to Cluster 1 showed a lower RNA expression of these TFs from Cluster 1’s subjects (*n* = 3) relative to control subjects (*n* = 2). Hence, differential methylation between MD clusters may have significant biological implications, and further investigation with complementary RNA-Seq data from the same sample cohort is warranted to further validate findings from this WGBS data. KEGG pathway analysis of unique TFs from the hypermethylated group also revealed an enrichment for pathways related to Th17, Th1, and Th2 differentiation. Previous studies have highlighted a strong connection with MD and underlying immune response [16]. For example, Fuse et al. 2003 have previously described an abnormal balance of Th1 and Th2 in MD patients by using a flow cytometry-based approach for measuring cytokines in peripheral blood of MD patients (*n* = 19). Hence, the differential DNA methylation in MD Cluster 1 from this study may be reflective of a similar cytokine profile that is lacking in MD Cluster 2 or 3 and warrants further investigation.

Significant DMCs were observed in Cluster 1 and Cluster 3 across the *KDMB4* gene, and this gene had significantly upregulated DNA accessibility in the ATAC-seq data. This is an interesting observation suggestive of an overall upregulation of *KDMB4* in MD irrespective of subcluster. *KDMB4* is a histone demethylase which impacts lysine 9 residue of histone 3 (H3K9me) and lysine 36 residue of histone 3 (H3K36me) specifically. H3K9me3 promotes heterochromatic states and in turn inhibits transcription. Among its various roles in lineage commitment and cell differentiation, H3K9me3 deposition has been previously shown to be involved in lineage commitment of Th2 lymphocytes by repressing Th1-specific loci through an SUV39H1-dependent pathway [17, 18]. Since transcription factors that were unique to MD Cluster 1 in our dataset showed a potential association with Th1 and Th2 cell differentiation pathways, further understanding of whether H3K9me3 deposition may be affecting essential molecular pathways related to MD subclusters is warranted. Among the key cellular processes in which H3K36me3 has been previously implicated are splicing regulation, DNA methylation, and DNA damage processes. In particular, H3K36me3 is thought to play key roles in maintaining neuronal development and has been associated with a variety of neurological disorders [19]. Gene Ontology enrichment of DMCs specific to MD Cluster 1 in our study also highlighted several pathways related to neuronal development. Hence, further understanding of whether H3K36me3 deposition may be affecting essential molecular pathways related to MD subclusters is warranted.

The neuronal-related pathways that seem to dominate genes from Cluster 1 DMCs may be related to cognitive performance in this Cluster compared to Cluster 2 or 3. For example, in previous studies, cognitive decline has been associated with MD duration, and vestibular dysfunction in particular has been identified as a modifiable risk factor following a structured treatment approach with patient education and lifestyle changes, as well as drug regimens of betahistine, diuretics, and steroids [20]. Although our study did not account for patient treatment and duration of disease, since MD patient subclusters have been observed across several different studies [4, 9–11], a longitudinal study where the effects of intervention on DNA methylation pre- and post-treatment may provide further insights on the molecular mechanisms associated with the different patient subclusters.

Finally, the observation of significant DMCs from Cluster 1, which were identified to overlap known splice sites from HEXEvents and were also in locations predicted to be where aberrant splicing occurs through the UCSC database, warrants further investigation. CD2 in particular is a surface antigen found on all peripheral blood T-cells, which mediates adhesion between T-cells and is implicated in the triggering of T-cells.

Interestingly, of the known hearing loss genes, only *DMXL2* was annotated to significant DMCs from both Cluster 1 versus Control and Cluster 3 versus Control (Supplementary Fig.  2 A, Supplementary Table 6). In addition, preliminary validation from qRT-PCR showed average DMXL2 expression was higher in all MD clusters compared to the control group, with Cluster 3 showing the greatest difference (17.2-fold-change) (Supplementary Fig. 3B). Furthermore, *DMXL2* has been highlighted in a previous study where MD patients were clustered based on their IL-1β levels [9]. Although a less stringent cut-off of 10% methylation difference was used to identify significant DMCs, WGBS of patients from this study also revealed significant DMCs in *DMXL2* irrespective of their IL-1β clustering. Rare variants in *DMXL2* have been attributed to autosomal dominant SNHL [21]. This gene encodes components of the rabconnectin protein complex which concentrates on synaptic vesicles and plays an essential role in neurosecretion [21]. Studies of zebrafish have localized the expression of *DMXL2* proteins to the basal regions of inner ear hair cells where synaptic vesicles are enriched [22]. GO pathways for synaptic signaling, trans-synaptic signaling, and chemical synaptic transmission were significantly enriched among hypomethylated DMCs associated with genes from Cluster 1 versus Cluster 2 (Fig. 1E, Fig. 2G). On the other hand, GO pathways for synapse organization were significantly enriched among hypomethylated DMCs associated with genes from Cluster 3 versus Control. Since *DMXL2* variants are already implicated in SNHL, aberrant DNA methylation of this gene identified from two independent WGBS studies, along with preliminary qRT-PCR results suggesting altered expression of this gene in MD clusters, implores further investigation of potential implications of *DMXL2* gene expression in MD with respect to DNA methylation-driven patient clusters.

## Conclusions

Together, the study provides associative evidence that DNA methylation may define MD patients into clusters with distinct molecular phenotypes. Differences related to neuronal development as well as Th1, Th2, and Th17 cell differentiation are more pronounced for Cluster 1 compared to Cluster 3, whereas Cluster 2 does not show a significant difference to healthy controls. We conclude that an underlying molecular phenotype that may be linked to immunity-related pathways, neuronal development pathways, as well as synaptic signaling pathways is an important consideration for understanding subgroups of MD patients which in turn may require specialized treatment options. These findings warrant further investigation through future studies where DNA methylation data is collected concurrent to cytokine profiles and transcriptomic data from the same MD patients.

## Methods

### Human subjects

We included a total of 40 patients with definite MD and 13 healthy controls that were recruited between January 2020 and April 2022 from Spanish referral centers. Patients were diagnosed according to the diagnostic criteria of the Barany Society for MD [23]. The experimental protocols (EPIVERT PI027-2020) of this study were approved by the Institutional Review Board in all participating hospitals, and every patient signed written informed consent. The study was carried out according to the principles of the Declaration of Helsinki revised in 2013 for investigation with humans. All human studies have been approved by the appropriate ethics committee.

### DNA extraction

DNA was extracted from peripheral blood mononuclear cells (PBMCs) using the QIAamp DNA Blood Mini Kit (Qiagen, Hilden, Germany), following the manufacturer’s protocol. DNA concentration and quality parameters were verified by Nanodrop (Thermo Fisher, Waltham, MA, USA) and Qubit (Invitrogen, Waltham, MA, USA) as previously described [9]. Additionally, DNA integrity was verified by electrophoresis in a 2% agarose gel. For WGBS, the minimum parameters considered were a concentration superior to 20 ng/µL, a 260/280 ratio superior to 1.8, and no observable smearing/DNA degradation by electrophoresis [24].

### WGBS library preparation

WGBS was carried out by Novogene (Oxford, UK). Briefly, Accel-NGS Methyl-Seq DNA Library Kit (Zymo Research, Irvine, CA, USA) was used to prepare NGS libraries from bisulphite-converted DNA for sequencing. For this, the samples were treated with bisulphite to convert the unmethylated cytosines to uracils, while retaining the methylated cytosines. This was followed by an adaptase step that performed tailing and ligation of truncated adapters to the 3′ ends. The extension and ligation steps added truncated adapters, which were followed by an indexing PCR step increasing the yield and incorporating full-length adapters for single or dual indexing. Finally, bead-based clean-ups removed oligonucleotides and small fragments [25].

### WGBS data analysis

Initial quality control, filtering, adapter trimming, and removal of methylation bias (m-bias) in the 5′ and 3′ ends of reads were conducted using FastQC v0.11.8 and Trim Galore v.0.4.2. M-bias guided the trimming of 9 bp from the 5′ ends and 3 bp from the 3′ ends. Quality assurance was performed using MultiQC. Quality-assured reads were then aligned to the human genome (hg38), deduplicated, examined for coverage, and extracted to a CpG count matrix using Bismark (v.0.22.1 https://github.com/FelixKrueger/Bismark). Bismark CpG coverage files were then imported into R (v.4.4.0 puppy cup) and converted to an rds object using the methylKit R package (v.0.99.2 https://github.com/al2na/methylKit) for downstream statistical processing.

### Differentially methylated CpG sites (DMC) analysis

The methylKit R package (v.0.99.2) was used to filter CpG sites with less than 10X coverage based on previously set recommendations [26]. CpG sites with methylation levels above 99.9% or below 0.001% were also removed from all samples. The methylation ratio was calculated as the number of C counts divided by the effective CT counts for each cytosine in CpG sites. Common base pair locations across all samples, after filtering, were then merged to obtain a methylbase object. All CpG sites on chromosomes X, Y, and M were also excluded from the downstream analysis. PCA analysis was used to filter outlier samples, and hierarchical clustering of samples was conducted using Ward’s Method with default parameters intrinsic to methylKit. DMCs were calculated using logistic regression, accounting for age and gender as covariates in the analysis. Significant DMCs were obtained using specific parameters, including *p*-value threshold of 0.05 and *q*-value threshold of 0.01, determined by the SLIM method. DMCs with at least a 25% difference in methylation between two conditions were considered significant.

### Gene ontology and pathway analysis for DMCs

Significant DMCs were annotated using the CAGEfightR package (v.1.16.0) with the TxDb Homo sapiens hg38 database of known genes (v.3.15.0). Gene lengths and GC content of these genes were obtained from Ensembl (https://asia.ensembl.org/index.html, release 112, May 2024). The resulting gene lists were then ranked by methylation scores, from highest to lowest weight. The methylation score for each gene was calculated based on GC content, gene length, and number of DMCs across the whole gene region, as shown in the following formula:

$$\frac{\Sigma |\text{Differential methylation}|}{\left(\text{GC content}\right)*(\text{Gene length})}$$

This formula assigned higher weights to shorter genes with a greater number of DMCs.

ClusterProfiler (v.4.4.4) was then used to perform the Gene Ontology (GO) analysis with the ranked gene list. Enriched GO pathways were calculated with 10,000 permutations, minimum of three genes per pathway, and a *p*-value cut-off of 0.05 [27]. For ease of visualization, only the top 10 pathways from each category with the lowest *p*-values were displayed.

### Deconvolution

Cell-type specific methylation markers were then used to deconvolute all samples for a theoretical assessment of cell type composition, as previously described [13] using wgbstools (v.0.2.0, https://github.com/nloyfer/wgbs_tools).

### Splice site identification

Genomic co-ordinates of all significant DMCs which overlapped the hg38 reference database of all known human genomic splice sites were identified in order to highlight genes that might be directly impacted by the presence of a DMC. Genomic co-ordinates of splice sites from each chromosome were downloaded from the database of human exon splicing events (HEXEvent (uci.edu), April 2024) and overlapping regions of significant DMCs from both Cluster 1 versus Control and Cluster 3 versus Control comparisons were identified using genomation (v.1.28.0). Constitutive exons from which splice sites were obtained were defined on the following criteria: alternative 3′ splice site, alternative 5′ splice site, as well as alternative 3′ and 5′ splice site simultaneously. An exon was defined as constitutive if it was alternatively spliced in at most 20% of ESTs.

### “Pseudo-bulk” ATAC sequencing

Single-cell peak coordinates and peak fragments cell metadata files were generated from FASTQ files that were aligned with the hg38 reference genome and analyzed using Cell Ranger ARC (v2.0.2). The MD samples used for this experiment were defined as IL-1β-driven autoinflammatory phenotype as previously described [4]. “Pseudo-bulk” analysis on merged IL-1β phenotype cluster MD samples was performed using “Merging Objects and” & “Visualization of Genomic Regions” pipeline from *Signac* (v1.12.0) [28]. *Signac*’s “scATAC-seq data integration” pipeline was used to integrate the data and correct for batch effects across samples. The FindMarkers function from Seurat (v.4.1.4) [20] was used for Differential Accessibility (DA) analysis, with a *p*-value adjustment threshold of 0.05 (Bonferroni correction) and a minimum log2 fold-change of ± 1.5, between MD samples and healthy controls.

### Differential methylation association with DNA accessibility

Significant DMCs identified between Cluster 1 and Control as well as Cluster 3 and Control analysis were further analyzed with respect to DNA accessibility identified from the pseudo-bulk ATAC-Seq analysis [14]. Genomic coordinates corresponding to significantly enriched DNA accessibility regions from *Seurat’s* FinMarkers analysis were then overlapped with genomic coordinates of significant DMCs from Cluster 1 versus Control as well as Cluster 3 versus Control using genomation (v.1.48.0).

### Transcription factor binding site (TFBS) identification (Homer)

Genomic co-ordinates of significant DMCs from Cluster 1 versus Control and Cluster 3 versus Control were converted to BED files with region lengths of ± 200 bp around each DMC. Transcription factor (TF) binding motifs enrichment analysis was then calculated with homermotifs.pl using the binomial distribution test intrinsic to the HOMER package (v 4.11, 10–24-2019) [29]. Significant TFs with *p*-value < 0.05 associated with significantly enriched TF binding motifs were obtained for Cluster 1 versus Control and Cluster 3 versus Control DMCs. KEGG pathway enrichment analysis was conducted using EnrichR [30] on the following sets of TF genes: (1) TFs that were unique to hypermethylated DMCs from Cluster 1 versus Control group and (2) TFs that were unique to hypomethylated DMCs from Cluster 1 versus Control group.

### Differential methylation association with hearing loss, stria vascularis, and axonal guidance gene sets

All genes which were annotated to DMCs from Cluster 1 versus Control as well as Cluster 3 versus Control were then compared against a known list of hearing loss genes retrieved from the deafness variation database (https://deafnessvariationdatabase.org/, accessed 2/07/2024) as has been described previously [9]. Similarly, a database of potential MD targets in adult stria vascularis (SV) identified previously from a metadata analysis [31] was used. The SV gene set (*n* = 834) was then overlapped with all genes which were annotated to DMCs from Cluster 1 versus Control as well as Cluster 3 versus Control. Finally, a previously curated list of genes (*n* = 234) which are known to affect axonal guidance in MD were overlapped with genes that were annotated to DMCs from Cluster 1 versus Control as well as Cluster 3 versus Control [15].

### Quantitative RT-PCR for validation

RNA was extracted with the High Pure RNA Isolation Kit (Roche), converted to cDNA using the Maxima First Strand cDNA Synthesis Kit (Thermo Fisher), and analyzed by SYBR Green real-time PCR using PerfeCTa SYBR Green FastMix ROX (Quanta). Primers used are listed in Supplementary Table 5.

## Supplementary Information

Below is the link to the electronic supplementary material.

ESM 1 Cell type composition analysis. (A) Heatmap showing unsupervised clustering of the various proportions of cell types detected in each sample using the deconvolution method. Cell type proportions are indicated in row names whereas sample clustering is indicated on the x-axis as are age and gender variables. (B) Clustered box plot showing the comparison of blood cell types across each sample specifically from deconvolution. The proportion of each cell type is indicated in the y-axis where each sample assessed (using Wilcox rank sum test) is indicated in the x-axis (PNG 289 KB)

High Resolution Image (TIF 21423 KB)

ESM 2 DMC overlaps with hearing loss and stria vascularis genes. (A) A Venn diagram showing the overlaps of all genes annotated to DMCs in Cluster 1 versus Control, DMCs in Cluster 3 versus Control as well as all known sensorineural hearing loss genes retrieved from Deafness variation database (https://deafnessvariationdatabase.org/, accessed on 02/07/2024). (B) A Venn diagram showing the overlaps of all genes annotated to DMCs in Cluster 1 versus Control, DMCs in Cluster 3 versus Control as well as all Meniere's Disease target genes in adult stria vascularis curated from a meta-analysis (31). (C) A table representing gene function retrieved from the Uniprot database, for all genes which were found to be significant in other WGBS studies of MD (9). (D) A Venn diagram showing the overlaps of all genes annotated to DMCs in Cluster 1 versus Control, DMCs in Cluster 3 versus Control as well as all genes known to affect axonal guidance in MD (LEUK_GeneSet and AGS_GeneSet). These datasets are the list of genes that showed a burden of missense variants in sporadic MD. The panel covered 263 different genes related with axonal guidance signalling (AGS) and leukocyte extravasation and cell adhesion pathways (LEUK)(15) (PNG 422 KB)

High Resolution Image (TIF 21423 KB)

ESM 3 Supplementary file3 Relative gene expression between MD clusters and control group. (A) A bar plot showing the average fold change (2^-ddCt) in expression of TFs within MD Cluster 1 RNA samples (n = 3) relative to the control RNA samples (n = 2). (B) A bar plot showing the average fold change (2^-ddCt) in expression of DMXL2 gene within MD Cluster 1 (n = 3), MD Cluster 2 (n = 4), MD Cluster 3 (n = 4) and control group (n = 2) (PNG 171 KB)

High Resolution Image (TIF 21423 KB)

Supplementary file4 (DOCX 38.4 KB )

## Data Availability

All data generated and analyzed during this study are included in this published article and can be found in Supplementary Table (large.xsl/.csv file to be uploaded through editor channels).

## References

[1] Perez-Carpena P, Lopez-Escamez JA (2020) Current understanding and clinical management of Meniere’s disease: a systematic review. Semin Neurol 40(1):138–150 31887752 10.1055/s-0039-3402065

[2] Parra-Perez AM, Lopez-Escamez JA (2023) Types of inheritance and genes associated with familial Meniere disease. J Assoc Res Otolaryngol: JARO 24(3):269–279 37022572 10.1007/s10162-023-00896-0 PMC10335989

[3] Frejo L, Requena T, Okawa S, Gallego-Martinez A, Martinez-Bueno M, Aran I et al (2017) Regulation of Fn14 receptor and NF-kappaB underlies inflammation in Meniere’s disease. Front Immunol 8:1739 29326686 10.3389/fimmu.2017.01739 PMC5733484

[4] Frejo L, Gallego-Martinez A, Requena T, Martin-Sanz E, Amor-Dorado JC, Soto-Varela A et al (2018) Proinflammatory cytokines and response to molds in mononuclear cells of patients with Meniere disease. Sci Rep 8(1):5974 29654306 10.1038/s41598-018-23911-4 PMC5899176

[5] Zhang N, Lyu Y, Guo J, Liu J, Song Y, Fan Z et al (2022) Bidirectional transport of IgE by CD23 in the inner ear of patients with Meniere’s disease. J Immunol 208(4):827–838 35046106 10.4049/jimmunol.2100745 PMC9012086

[6] Lopez-Escamez JA, Vela J, Frejo L (2023) Immune-related disorders associated with Meniere’s disease: a systematic review and meta-analysis. Otolaryngol Head Neck Surg 169(5):1122–1131 37272729 10.1002/ohn.386

[7] Gazquez I, Soto-Varela A, Aran I, Santos S, Batuecas A, Trinidad G et al (2011) High Prevalence of Systemic Autoimmune Diseases in Patients with Menière’s Disease. PLoS One 6(10):e26759 22053211 10.1371/journal.pone.0026759 PMC3203881

[8] Vambutas A, Pathak S (2016) AAO: Autoimmune and autoinflammatory (disease) in otology: what is new in immune-mediated hearing loss. Laryngoscope Investig Otolaryngol 1(5):110–115 27917401 10.1002/lio2.28 PMC5113311

[9] Flook M, Escalera-Balsera A, Gallego-Martinez A, Espinosa-Sanchez JM, Aran I, Soto-Varela A et al (2021) DNA methylation signature in mononuclear cells and proinflammatory cytokines may define molecular subtypes in sporadic Meniere disease. Biomedicines 9(11):1530 34829759 10.3390/biomedicines9111530 PMC8615058

[10] Flook M, Rojano E, Gallego-Martinez A, Escalera-Balsera A, Perez-Carpena P, Moleon MDC et al (2024) Cytokine profiling and transcriptomics in mononuclear cells define immune variants in Meniere disease. Genes Immun 25(2):124–131 38396174 10.1038/s41435-024-00260-z PMC11023934

[11] Flook M, Escalera-Balsera A, Rybakowska P, Frejo L, Batuecas-Caletrio A, Amor-Dorado JC et al (2023) Single-cell immune profiling of Meniere disease patients. Clin Immunol 252:109632 37178857 10.1016/j.clim.2023.109632

[12] Moore LD, Le T, Fan G (2013) DNA methylation and its basic function. Neuropsychopharmacology 38(1):23–38 22781841 10.1038/npp.2012.112 PMC3521964

[13] Loyfer N, Magenheim J, Peretz A, Cann G, Bredno J, Klochendler A et al (2023) A DNA methylation atlas of normal human cell types. Nature 613(7943):355–364 36599988 10.1038/s41586-022-05580-6 PMC9811898

[14] Cruz-Granados P, Frejo L, Perez-Carpena P, Amor-Dorado JC, Dominguez-Duran E, Fernandez-Nava MJ et al (2024) Multiomic-based immune response profiling in migraine, vestibular migraine and Meniere’s disease. Immunology 173(4):768–779 39294737 10.1111/imm.13863

[15] Gallego-Martinez A, Requena T, Roman-Naranjo P, May P, Lopez-Escamez JA (2020) Enrichment of damaging missense variants in genes related with axonal guidance signalling in sporadic Meniere’s disease. J Med Genet 57(2):82–88 31494579 10.1136/jmedgenet-2019-106159

[16] Fuse T, Hayashi T, Oota N, Fukase S, Asano S, Kato T et al (2003) Immunological responses in acute low-tone sensorineural hearing loss and Meniere’s disease. Acta Otolaryngol 123(1):26–31 12625569 10.1080/0036554021000028074

[17] Ninova M, Fejes Toth K, Aravin AA (2019) The control of gene expression and cell identity by H3K9 trimethylation. Development 146(19):dev181180 31540910 10.1242/dev.181180 PMC6803365

[18] Allan RS, Zueva E, Cammas F, Schreiber HA, Masson V, Belz GT et al (2012) An epigenetic silencing pathway controlling T helper 2 cell lineage commitment. Nature 487(7406):249–253 22763435 10.1038/nature11173

[19] Zaghi M, Broccoli V, Sessa A (2019) H3K36 methylation in neural development and associated diseases. Front Genet 10:1291 31998360 10.3389/fgene.2019.01291 PMC6962298

[20] Zhong J, Li X, Xu J, Chen W, Gao J, Lu X et al (2023) Analysis of cognitive function and its related factors after treatment in Meniere’s disease. Front Neurosci 17:1137734 37081934 10.3389/fnins.2023.1137734 PMC10112666

[21] Chen DY, Liu XF, Lin XJ, Zhang D, Chai YC, Yu DH et al (2017) A dominant variant in DMXL2 is linked to nonsyndromic hearing loss. Genet Med: Off J Am College Med Genet 19(5):553–558 10.1038/gim.2016.142 27657680

[22] Einhorn Z, Trapani JG, Liu Q, Nicolson T (2012) Rabconnectin3alpha promotes stable activity of the H+ pump on synaptic vesicles in hair cells. J Neurosci: Off J Soc Neurosci 32(32):11144–11156 10.1523/JNEUROSCI.1705-12.2012 PMC3428958 22875945

[23] Lopez-Escamez JA, Carey J, Chung WH, Goebel JA, Magnusson M, Mandala M et al (2015) Diagnostic criteria for Meniere’s disease. J Vestib Res 25(1):1–7 25882471 10.3233/VES-150549

[24] Szczepek AJ, Frejo L, Vona B, Trpchevska N, Cederroth CR, Caria H et al (2019) Recommendations on collecting and storing samples for genetic studies in hearing and tinnitus research. Ear Hear 40(2):219–226 29889665 10.1097/AUD.0000000000000614 PMC6400449

[25] Olova N, Krueger F, Andrews S, Oxley D, Berrens RV, Branco MR et al (2018) Comparison of whole-genome bisulfite sequencing library preparation strategies identifies sources of biases affecting DNA methylation data. Genome Biol 19(1):33 29544553 10.1186/s13059-018-1408-2 PMC5856372

[26] Ziller MJ, Hansen KD, Meissner A, Aryee MJ (2015) Coverage recommendations for methylation analysis by whole-genome bisulfite sequencing. Nat Methods 12(3):230–232 (**1 p following 2**) 25362363 10.1038/nmeth.3152 PMC4344394

[27] Wu T, Hu E, Xu S, Chen M, Guo P, Dai Z et al (2021) ClusterProfiler 40: a universal enrichment tool for interpreting omics data. Innovation (Camb). 2(3):100141 34557778 10.1016/j.xinn.2021.100141 PMC8454663

[28] Stuart T, Srivastava A, Madad S, Lareau CA, Satija R (2021) Single-cell chromatin state analysis with signac. Nat Methods 18(11):1333–1341 34725479 10.1038/s41592-021-01282-5 PMC9255697

[29] Heinz S, Benner C, Spann N, Bertolino E, Lin YC, Laslo P et al (2010) Simple combinations of lineage-determining transcription factors prime cis-regulatory elements required for macrophage and B cell identities. Mol Cell 38(4):576–589 20513432 10.1016/j.molcel.2010.05.004 PMC2898526

[30] Xie Z, Bailey A, Kuleshov MV, Clarke DJB, Evangelista JE, Jenkins SL et al (2021) Gene set knowledge discovery with enrichr. Curr Protoc 1(3):e90 33780170 10.1002/cpz1.90 PMC8152575

[31] Gu S, Olszewski R, Nelson L, Gallego-Martinez A, Lopez-Escamez JA, Hoa M (2021) Identification of potential Meniere’s disease targets in the adult stria vascularis. Front Neurol 12:630561 33613436 10.3389/fneur.2021.630561 PMC7894210

