# Supplementary figures and images for: Whole genome DNA methylation profiles define Meniere’s disease subclusters
Source: J Mol Med (Berl). 2025 Aug 6;103(10):1191–204. doi: 10.1007/s00109-025-02581-6 (PMC12449340; doi:10.1007/s00109-025-02581-6)

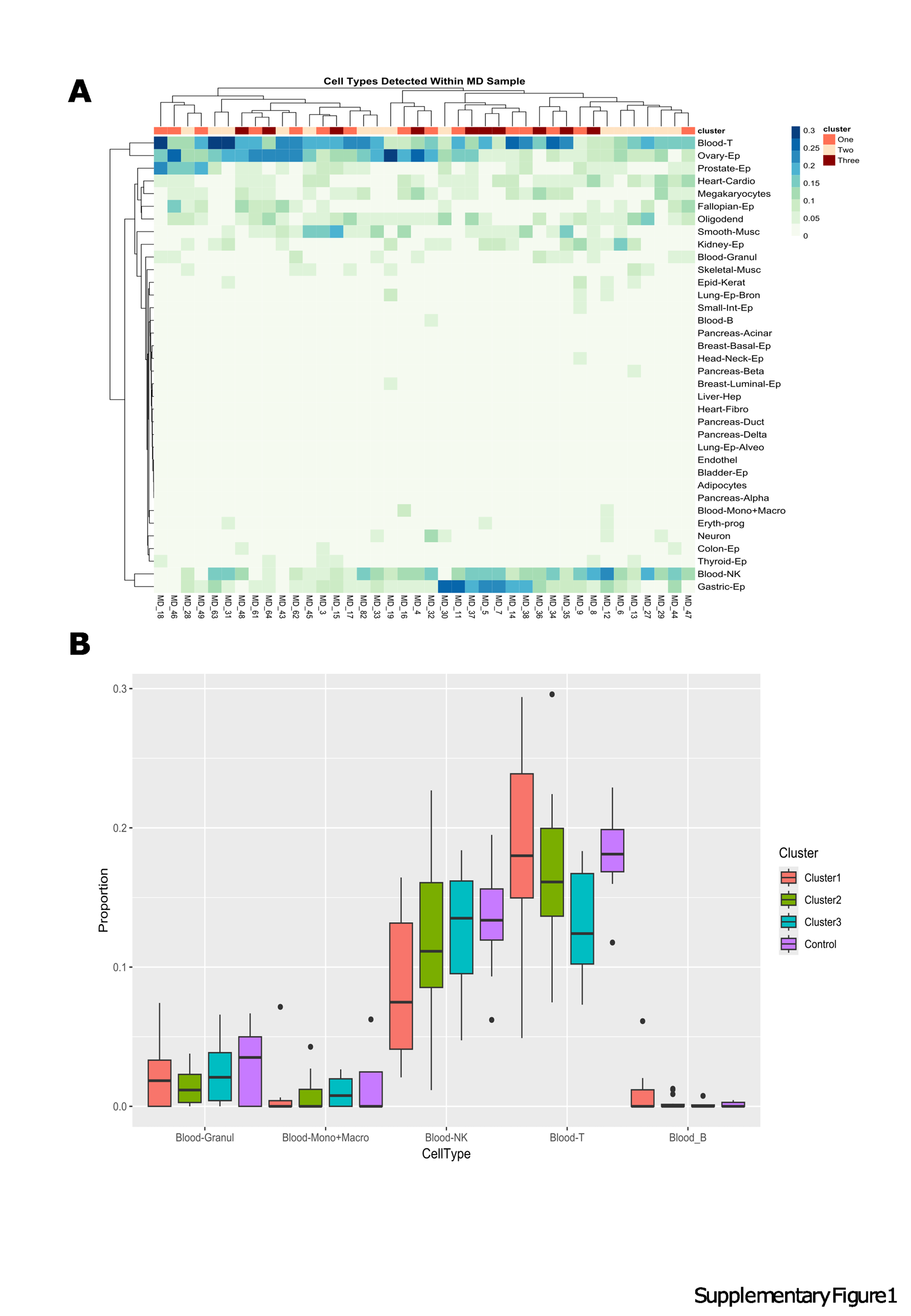

Supplement: Supplementary file 1 — Cell type composition analysis. (A) Heatmap showing unsupervised clustering of the various proportions of cell types detected in each sample using the deconvolution method. Cell type proportions are indicated in row names whereas sample clustering is indicated on the x-axis as are age and gender variables. (B) Clustered box plot showing the comparison of blood cell types across each sample specifically from deconvolution. The proportion of each cell type is indicated in the y-axis where each sample assessed (using Wilcox rank sum test) is indicated in the x-axis (PNG 289 KB) [file 109_2025_2581_Fig4_ESM.png]

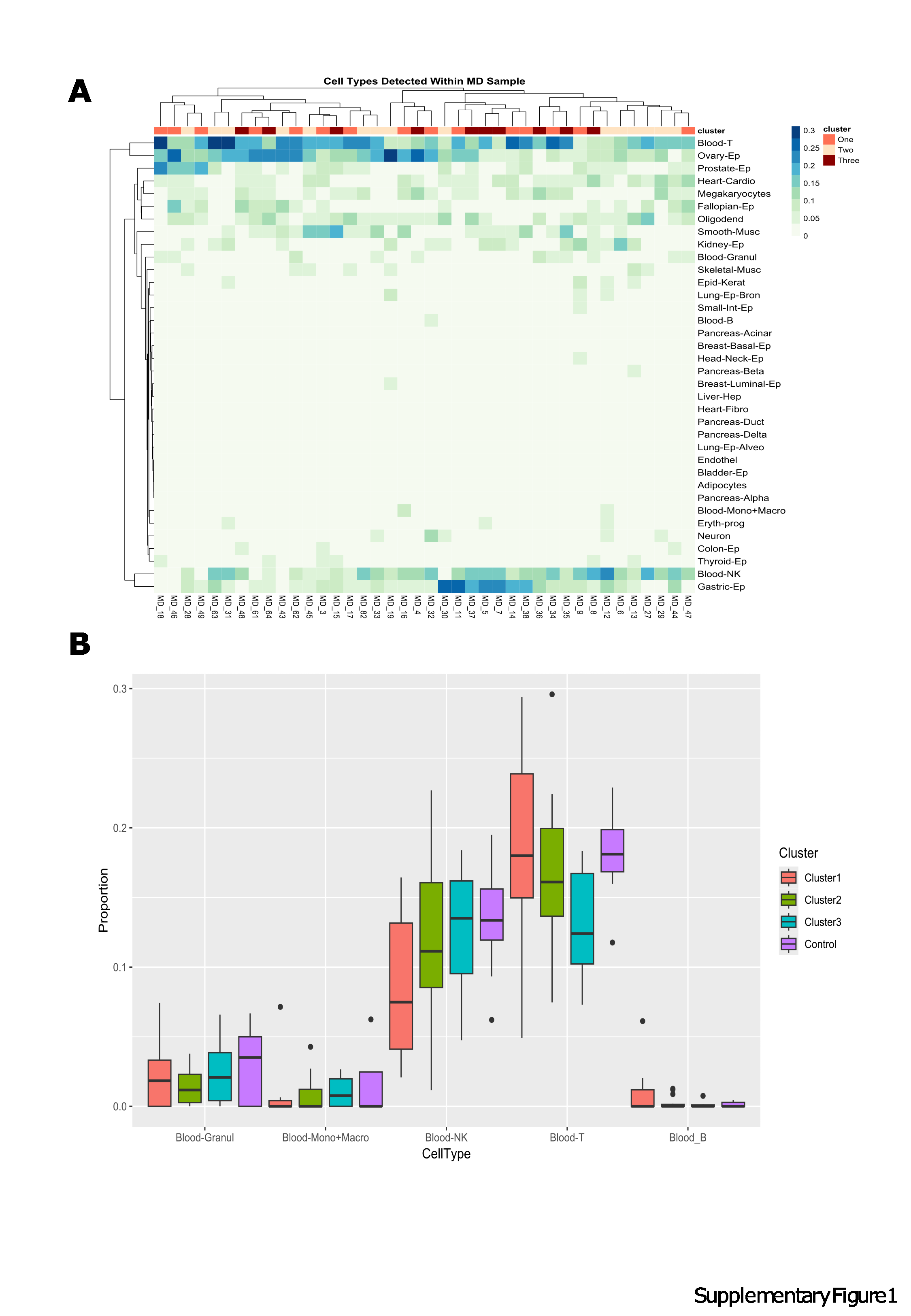

Supplement: Supplementary file 2 — High Resolution Image (TIF 21423 KB) [file 109_2025_2581_MOESM1_ESM.tiff]

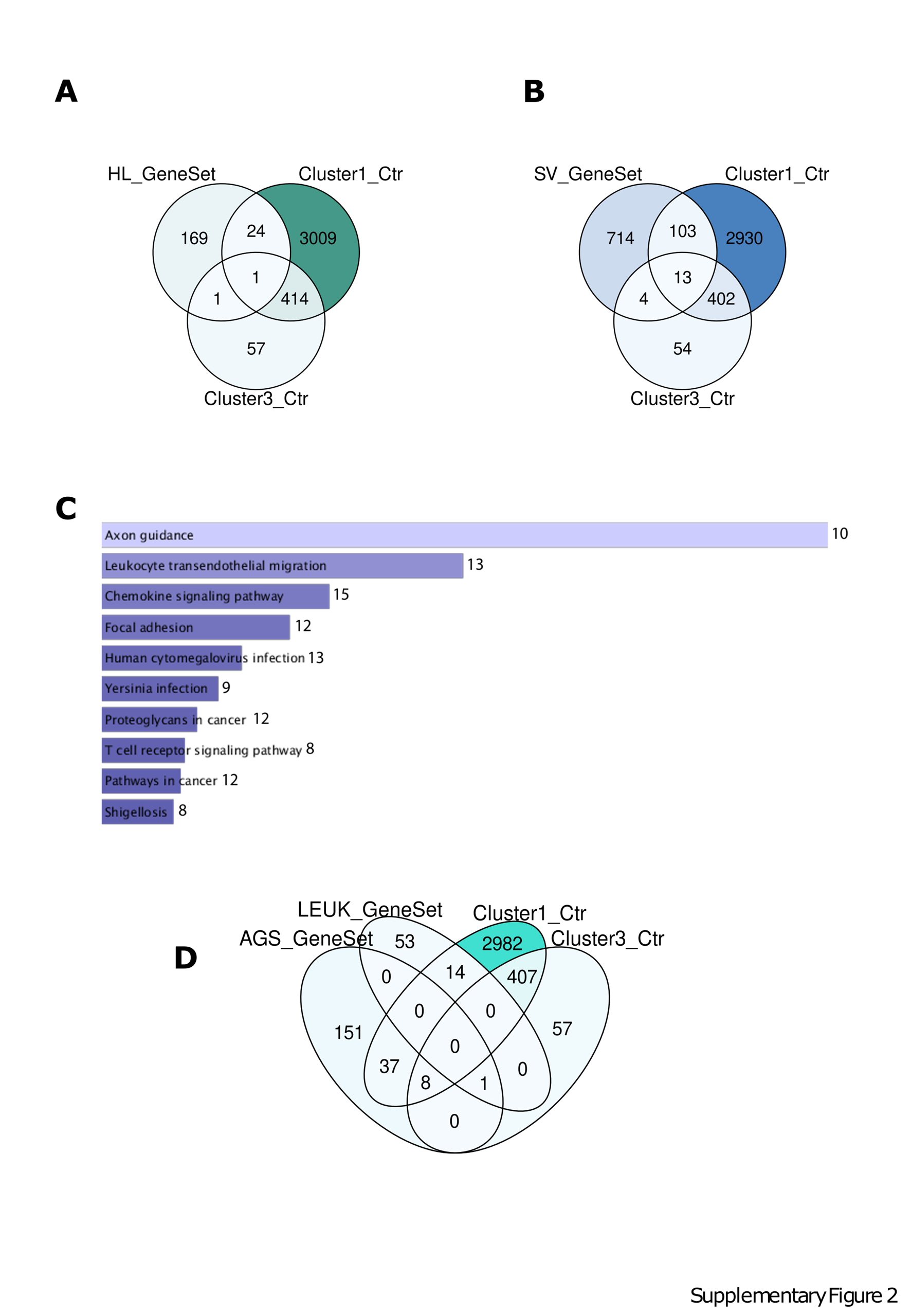

Supplement: Supplementary file 3 — DMC overlaps with hearing loss and stria vascularis genes. (A) A Venn diagram showing the overlaps of all genes annotated to DMCs in Cluster 1 versus Control, DMCs in Cluster 3 versus Control as well as all known sensorineural hearing loss genes retrieved from Deafness variation database (https://deafnessvariationdatabase.org/, accessed on 02/07/2024). (B) A Venn diagram showing the overlaps of all genes annotated to DMCs in Cluster 1 versus Control, DMCs in Cluster 3 versus Control as well as all Meniere's Disease target genes in adult stria vascularis curated from a meta-analysis (31). (C) A table representing gene function retrieved from the Uniprot database, for all genes which were found to be significant in other WGBS studies of MD (9). (D) A Venn diagram showing the overlaps of all genes annotated to DMCs in Cluster 1 versus Control, DMCs in Cluster 3 versus Control as well as all genes known to affect axonal guidance in MD (LEUK_GeneSet and AGS_GeneSet). These datasets are the list of genes that showed a burden of missense variants in sporadic MD. The panel covered 263 different genes related with axonal guidance signalling (AGS) and leukocyte extravasation and cell adhesion pathways (LEUK)(15) (PNG 422 KB) [file 109_2025_2581_Fig5_ESM.png]

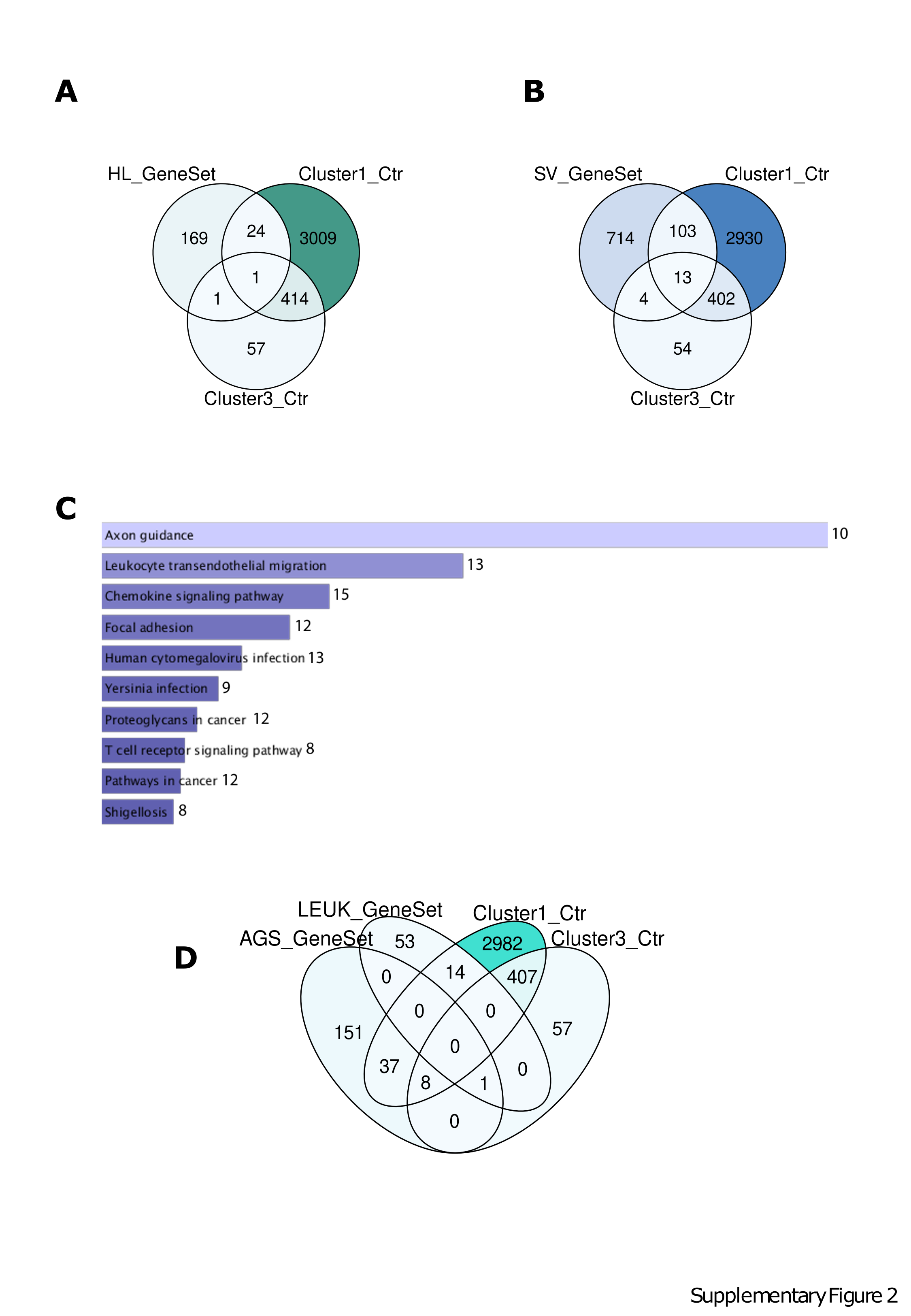

Supplement: Supplementary file 4 — High Resolution Image (TIF 21423 KB) [file 109_2025_2581_MOESM2_ESM.tiff]

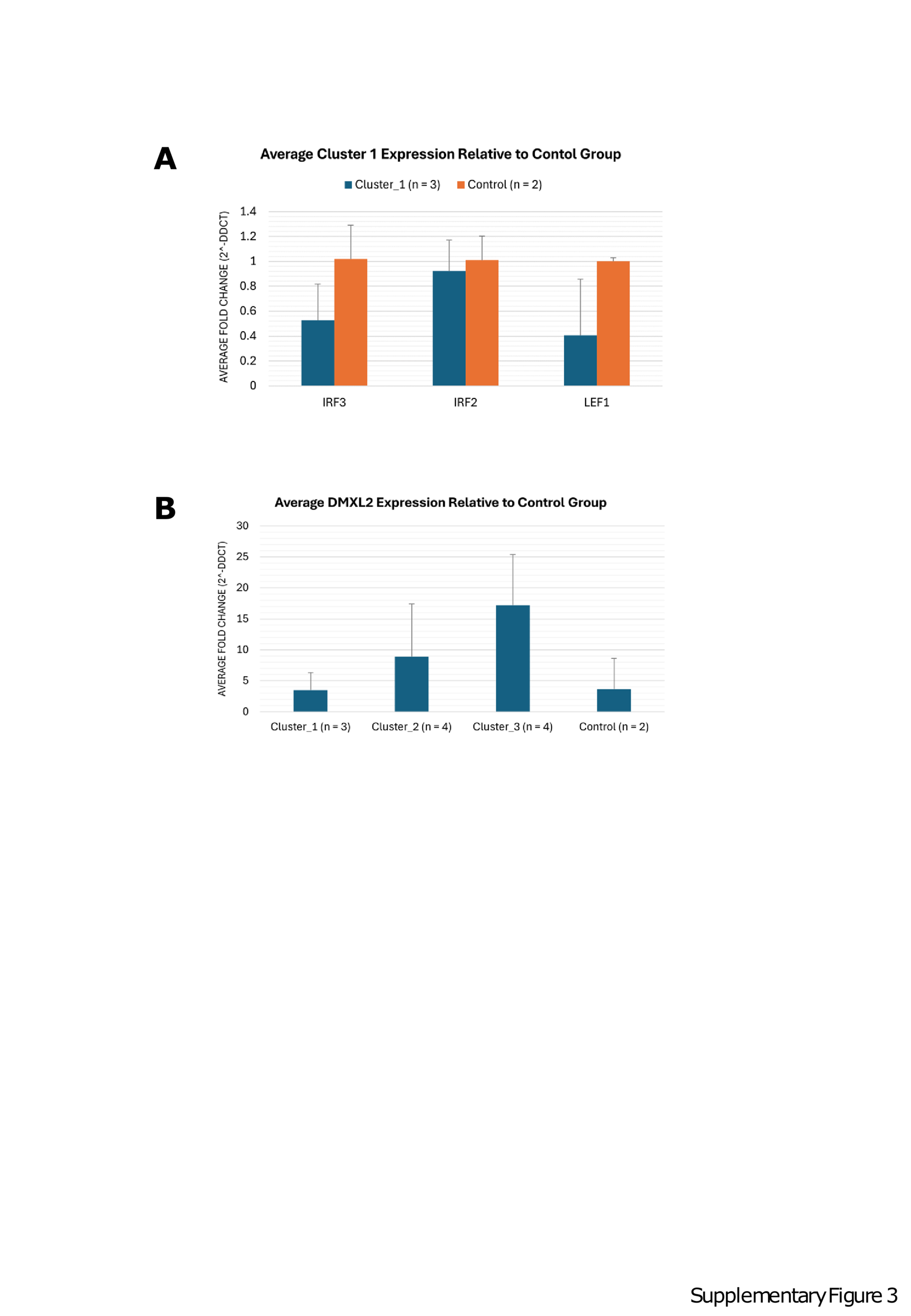

Supplement: Supplementary file 5 — Supplementary file3 Relative gene expression between MD clusters and control group. (A) A bar plot showing the average fold change (2^-ddCt) in expression of TFs within MD Cluster 1 RNA samples (n = 3) relative to the control RNA samples (n = 2). (B) A bar plot showing the average fold change (2^-ddCt) in expression of DMXL2 gene within MD Cluster 1 (n = 3), MD Cluster 2 (n = 4), MD Cluster 3 (n = 4) and control group (n = 2) (PNG 171 KB) [file 109_2025_2581_Fig6_ESM.png]

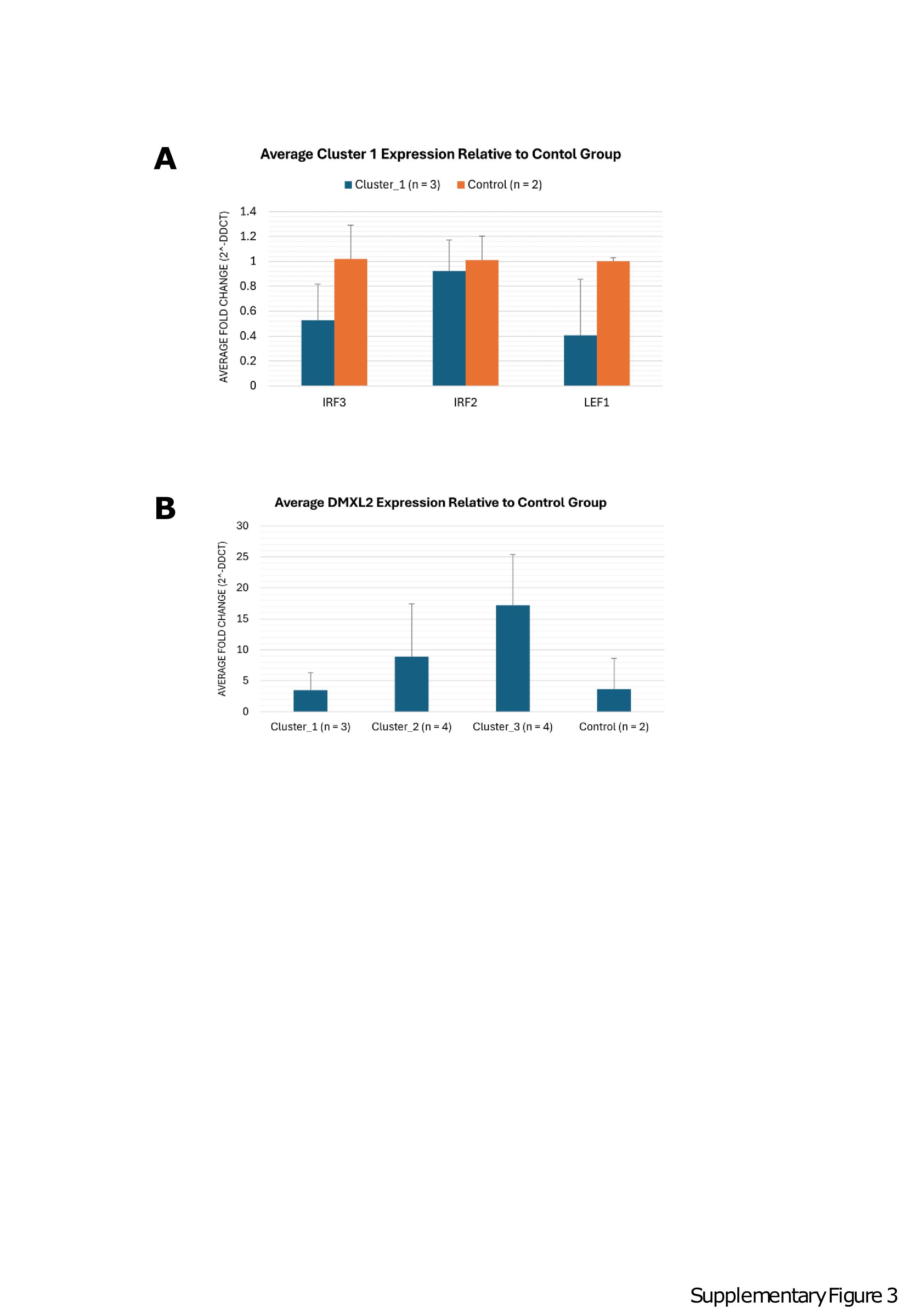

Supplement: Supplementary file 6 — High Resolution Image (TIF 21423 KB) [file 109_2025_2581_MOESM3_ESM.tiff]
