# Supplementary material for: Whole genome DNA methylation profiles define Meniere’s disease subclusters
Source: J Mol Med (Berl). 2025 Aug 6;103(10):1191–204. doi: 10.1007/s00109-025-02581-6 (PMC12449340; doi:10.1007/s00109-025-02581-6)
Supplement: Supplementary file 7 — Supplementary file4 (DOCX 38.4 KB ) [file 109_2025_2581_MOESM4_ESM.docx]

| **Cluster1_HypermethylatedDMCs** | **Cluster1_HypomethylatedDMCs** |
| --- | --- |
| IRF3 | ETS |
| IRF2 | SPDEF |
| LEF1 | E2F6 |
| IRF:BATF | CEBP:CEBP |
| IRF4 | CREB5 |
| Tcf3 | Gfi1b |
| JunB | Atf7 |
| BATF | NFE2L2 |
| Oct-02 | HIF-1a |
| Oct-06 | Foxo1 |
| Fos | ETS:RUNX |
| AP-1 | NFAT:AP1 |
| Fra1 | GLIS3 |
| Oct-11 | Bach1 |
| GATA | ETS:E-box |
| Fra2 | Nrf2 |
| Egr1 | E2F3 |
| Atf3 | DMRT1 |
| Egr2 | NF-E2 |
| Oct-04 |  |
| TCFL2 |  |
| Brn1 |  |
| Gata6 |  |
| GATA3 |  |
| Gata4 |  |
| Gata2 |  |
| Fosl2 |  |
| Hoxa10 |  |
| Npas4 |  |
| Tbx21 |  |
| Gata1 |  |
| WT1 |  |
| Sox10 |  |
| Jun-AP1 |  |

**Supplementary Table 1:** A table representing transcription factors annotated to genomic regions +/-200bp of hypermethylated and hypomethylated DMCs which are unique to cluster 1 vs control group.

| **Gene Name** | **Function** |
| --- | --- |
| *DMXL2* | May serve as a scaffold protein for MADD and RAB3GA on synaptic vesicles (2). |
| *ADGRV1* | Required for the hair bundle ankle formation, which connects growing stereocilia in developing cochlear hair cells of the inner ear. Enhances the stability of this protein in myelin-forming cells of the auditory pathway. |
| *MSRB1* | Catalyzes the reduction of free and protein-bound methionine sulfoxide to methionine. Isoform 2 is essential for hearing. |

**Supplementary Table 2:** A table representing gene function retrieved from the Uniprot database, for all genes which were found to be relevant in other WGBS studies of MD.

| **SVGenes_Cluster1** | **SVGenes_Cluster3** | **HL_Cluster1** | **HL_Cluster3** |
| --- | --- | --- | --- |
| *ABL1* | ADD3 | BTD | DMXL2 |
| *ABLIM1* | DMXL2 | CDC14A |  |
| *ABLIM2* | FYN | CEP78 |  |
| *ACTN1* | GNG7 | CHD7 |  |
| *ACTR3* | KMT2C | CHSY1 |  |
| *ADAM23* | MTR | COL11A1 |  |
| *ADAMTS9* | P2RX7 | DCDC2 |  |
| *ADD1* | PAK1 | DIAPH1 |  |
| *ADD3* | PDGFC | DMXL2 |  |
| *ANK2* | PPP3CA | EYA1 |  |
| *ARNT2* | PPP3R1 | GRXCR2 |  |
| *BCL11B* | PTPN22 | HOMER2 |  |
| *BMPR1B* | ROCK2 | KMT2D |  |
| *BMPR2* | SEMA3A | LOXL3 |  |
| *C3AR1* | SGK1 | MANBA |  |
| *CACNA2D1* | UBE2L3 | MET |  |
| *CCR3* |  | MSRB3 |  |
| *CDC14A* |  | NLRP3 |  |
| *CDHR4* |  | OPA1 |  |
| *CLDN10* |  | PDE1C |  |
| *COL11A1* |  | PLS1 |  |
| *COL26A1* |  | RDX |  |
| *CTLA4* |  | SLC33A1 |  |
| *CTNNA1* |  | SPATA5 |  |
| *CTNNB1* |  | TJP2 |  |
| *DCDC2* |  |  |  |
| *DIAPH1* |  |  |  |
| *DMXL2* |  |  |  |
| *DPT* |  |  |  |
| *DTNA* |  |  |  |
| *F11R* |  |  |  |
| *FAM107B* |  |  |  |
| *FYN* |  |  |  |
| *FZD3* |  |  |  |
| *GLI3* |  |  |  |
| *GNAI1* |  |  |  |
| *GNAI3* |  |  |  |
| *GNG2* |  |  |  |
| *GNG7* |  |  |  |
| *GPR55* |  |  |  |
| *GRHL2* |  |  |  |
| *GRXCR2* |  |  |  |
| *GSK3B* |  |  |  |
| *HOMER2* |  |  |  |
| *HTR2A* |  |  |  |
| *IL1R1* |  |  |  |
| *ITK* |  |  |  |
| *KMT2C* |  |  |  |
| *MAPK1* |  |  |  |
| *MAPK10* |  |  |  |
| *MCUR1* |  |  |  |
| *MET* |  |  |  |
| *MME* |  |  |  |
| *MSR1* |  |  |  |
| *MSRB3* |  |  |  |
| *NCK1* |  |  |  |
| *NFATC3* |  |  |  |
| *NKAIN2* |  |  |  |
| *NLRP3* |  |  |  |
| *NOL3* |  |  |  |
| *NPR3* |  |  |  |
| *NTSR1* |  |  |  |
| *OTOGL* |  |  |  |
| *P2RX7* |  |  |  |
| *PAK1* |  |  |  |
| *PARD3* |  |  |  |
| *PDE1C* |  |  |  |
| *PDGFC* |  |  |  |
| *PECAM1* |  |  |  |
| *PIK3C2G* |  |  |  |
| *PIK3CA* |  |  |  |
| *PKD2* |  |  |  |
| *PLXNC1* |  |  |  |
| *PPP3CA* |  |  |  |
| *PPP3CB* |  |  |  |
| *PPP3R1* |  |  |  |
| *PRKCB* |  |  |  |
| *PRKG1* |  |  |  |
| *PXN* |  |  |  |
| *RAC1* |  |  |  |
| *RAP1A* |  |  |  |
| *RAP1B* |  |  |  |
| *RASA1* |  |  |  |
| *RDX* |  |  |  |
| *ROCK1* |  |  |  |
| *ROCK2* |  |  |  |
| *ROR1* |  |  |  |
| *ROR2* |  |  |  |
| *RYK* |  |  |  |
| *SCN7A* |  |  |  |
| *SDC2* |  |  |  |
| *SEMA3A* |  |  |  |
| *SEMA3C* |  |  |  |
| *SEMA4B* |  |  |  |
| *SEMA4D* |  |  |  |
| *SEMA5A* |  |  |  |
| *SGK1* |  |  |  |
| *SLC12A1* |  |  |  |
| *SLC24A3* |  |  |  |
| *SLC2A3* |  |  |  |
| *SLC44A2* |  |  |  |
| *SLC8A1* |  |  |  |
| *SLC8A3* |  |  |  |
| *SLIT2* |  |  |  |
| *SRGAP1* |  |  |  |
| *SRGAP3* |  |  |  |
| *STAT1* |  |  |  |
| *TJP2* |  |  |  |
| *TNFSF11* |  |  |  |
| *TPTE2P6* |  |  |  |
| *TRPC6* |  |  |  |
| *UNC5C* |  |  |  |
| *USP3* |  |  |  |
| *VAV3* |  |  |  |
| *VCL* |  |  |  |
| *WIPF1* |  |  |  |

**Supplementary Table 3**: A table showing genes from Cluster 1 Vs Control DMCs and Cluster 3 Vs Control DMCs which overlap with stria vascularis (SV) genes identified from a meta-analysis (Gu S, Olszewski R, Nelson L, Gallego-Martinez A, Lopez-Escamez JA, Hoa M. Identification of Potential Meniere's Disease Targets in the Adult Stria Vascularis. Front Neurol. 2021;12:630561) as well as hearing loss (HL) genes identified from the sensorineural hearing loss database (https://deafnessvariationdatabase.org/, accessed 2/07/2024). Green cells indicate overlaps between Cluster 1, Cluster 3 and SV genes. Blue cells indicate overlaps between Cluster 1, Cluster 3 and HL genes.

| **AGSGenes_Cluster1** | **LEUKGenes_Cluster1** |
| --- | --- |
| *ABLIM1* | *CTNNA1* |
| *ACTR3* | *F11R* |
| *ADAM23* | *CLDN10* |
| *ADAMTS9* | *VAV3* |
| *GLI3* | *MAPK10* |
| *GNAI1* | *PECAM1* |
| *GNG2* | *CTNNB1* |
| *MME* | *PXN* |
| *SDC2* | *ACTN1* |
| *SEMA3C* | *VCL* |
| *SEMA5A* | *ITK* |
| *SLIT2* | *PRKCB* |
| *UNC5C* | *RAP1A* |
| *WIPF1* | *RAP1B* |
| *TRPC6* |  |
| *PPP3CB* |  |
| *NFATC3* |  |
| *RAC1* |  |
| *ABLIM2* |  |
| *NCK1* |  |
| *ROCK1* |  |
| *ABL1* |  |
| *RASA1* |  |
| *MAPK1* |  |
| *GNAI3* |  |
| *SRGAP1* |  |
| *SRGAP3* |  |
| *GSK3B* |  |
| *SEMA4D* |  |
| *SEMA4B* |  |
| *PLXNC1* |  |
| *FZD3* |  |
| *RYK* |  |
| *PIK3CA* |  |
| *PARD3* |  |
| *BMPR2* |  |
| *BMPR1B* |  |

**Supplementary Table 4:** A table showing genes from Cluster 1 Vs Control DMCs which uniquely overlap with previously curated genes known to be linked with axonal guidance in MD. AGS, axonal guidance signaling; LEUK, leukocyte extravasation and cell adhesion pathways. No genes unique to Cluster 3 Vs Control DMCs were found to overlap this gene set.

| **Gene** | **Forward (5’ - 3’)** | **Reverse (5’ - 3’)** |
| --- | --- | --- |
| IRF3 | GACCTTCCATCGTAGGCCG | ACGTAGCGCATCACTCCCCT |
| IRF2 | TCCTGAGTATGCGGTCCTGA | TCAGTCGTTTCGCTTTCTGC |
| LEF1 | TATCCCAATGGCAGAGGTGG | TCGAGTAGGAGGGTCCCTTG |
| DMXL2 | GGTTGAAGCTGATCTGGGCT | CATCCGCAACACCACACTTG |

**Supplementary Table 5:** List of primer sequences used to amplify cDNA for quantitative assessment of relative gene expression.

| **Chromosome** | **Location** | **pvalue** | **qvalue** | **Methylation (%)** |
| --- | --- | --- | --- | --- |
| chr15 | 51586220 | 7.79E-16 | 2.65E-13 | -40.61316288 |
| chr15 | 51586194 | 2.17E-15 | 6.90E-13 | -43.03042763 |
| chr15 | 51586440 | 4.46E-14 | 1.10E-11 | -25.87600112 |
| chr15 | 51586431 | 2.08E-13 | 4.55E-11 | -27.68957094 |

**Supplementary Table 6:** The genomic position, significance and location of DMCs detected in DMXL2.

Additional references

1. Cruz-Granados P, Frejo L, Perez-Carpena P, Amor-Dorado JC, Dominguez-Duran E, Fernandez-Nava MJ, et al. Multiomic-based immune response profiling in migraine, vestibular migraine and Meniere's disease. Immunology. 2024;173(4):768-79.

2. Chen DY, Liu XF, Lin XJ, Zhang D, Chai YC, Yu DH, et al. A dominant variant in DMXL2 is linked to nonsyndromic hearing loss. Genetics in medicine : official journal of the American College of Medical Genetics. 2017;19(5):553-8.

3. Gu S, Olszewski R, Nelson L, Gallego-Martinez A, Lopez-Escamez JA, Hoa M. Identification of Potential Meniere's Disease Targets in the Adult Stria Vascularis. Front Neurol. 2021;12:630561.
